# Mode-Aware Radio Resource Allocation Algorithm in Hybrid Users Based Cognitive Radio Networks

**DOI:** 10.3390/s25165086

**Published:** 2025-08-15

**Authors:** Sirui Luo, Ziwei Chen

**Affiliations:** 1 Centre for Advanced Spatial Analysis, University College London, Gower Street, London WC1E 6BT, UK; sirui.luo.24@ucl.ac.uk; 2 Department of Electronics, Beijing Jiaotong University, Beijing 100044, China

**Keywords:** cognitive radio network, energy efficiency, hybrid users, radio resource allocation, user fairness

## Abstract

In cognitive radio networks (CRNs), primary users (*PU*s) have the highest priority in channel resource allocation. Secondary users (*SU*s) can generally only utilize temporarily unused channels of *PU*s, share channels with *PU*s, or cooperate with *PU*s to gain priority through the interweave, underlay, and overlay modes. Traditional optimization schemes for channel resource allocation often lead to structural wastage of channel resources, whereas approaches such as reinforcement learning—though effective—require high computational power and thus exhibit poor adaptability in industrial deployments. Moreover, existing works typically optimize a single performance metric with limited scenario scalability. To address these limitations, this paper proposes a CR network algorithm based on the hybrid users (HU) concept, which links the Interweave and Underlay modes through an adaptive threshold for mode switching. The algorithm employs the Hungarian method for *SU* channel allocation and applies a multi-level power adjustment strategy when *PU*s and *SU*s share the same channel to maximize channel resource utilization. Simulation results under various parameter settings show that the proposed algorithm improves the average signal to interference plus noise ratio (SINR) of *SU*s while ensuring *PU* service quality, significantly enhances network energy efficiency, and markedly improves Jain’s fairness among *SU*s in low-power scenarios.

## 1. Introduction

Against the backdrop of increasingly tight wireless spectrum resources, about 50 billion smart devices are expected to access IoT systems worldwide by 2030 [1]. However, constrained by limited spectrum resources and increasingly complex heterogeneous communication scenarios, current fifth-generation (5G) mobile communication technologies (despite already having state-of-the-art performance) still struggle to meet future demands [1,2]. To enhance the efficiency of spectrum utilization, cognitive radio networks (CRNs) are considered a cutting-edge solution. In CRNs, primary users (*PU*s) and secondary users (*SU*s) belong to the primary network (PN) and secondary network (SN), respectively, and their behavioral modes include interweave mode (IM), underlay mode (UM), and overlay mode (OM) [2,3]. In IM mode, *SU*s utilize the *PU*s only when they are idle in the “spectrum hole” for transmission, and the strategy is dynamic spectrum access (DSA). In UM mode, the *SU* coexists with the *PU* and transmits using spectrum sharing (SS), which is required to keep the interference to the *PU* within a threshold [3]. In OM mode, the *SU* helps the *PU* to transmit through collaborative communication or precoding, thus gaining the transmission of its own information [4].

Early optimization in CRNs predominantly relied on time division multiple access (TDMA) and frequency division multiple access (FDMA) resource allocation schemes; however, these methods have been shown to cause structural inefficiencies in time–frequency resource utilization in fourth-generation (4G) systems [5,6]. In recent years, CRNs’ resource optimization has been divided into three main classes of methods: The first is mathematical optimization, such as Karush–Kuhn–Tucker conditions (KKT) [7], Lagrangian dyadic and convex optimization methods [2], which transform the problem into an optimization or (pseudo) convex problem-solving process [8]. The second category includes heuristic algorithms, such as the genetic algorithm (GA) [9], particle swarm optimization (PSO) [10], ant colony optimization (ACO) [11,12,13], and the whale optimization algorithm (WOA) [14]. The third category is reinforcement learning (RL)-based methods, such as Q-learning [1], which utilize Markov Decision Processes (MDPs) for intelligence training [15,16,17]. Recent works have further explored RL-based and joint optimization strategies for NOMA resource allocation, including multi-agent deep reinforcement learning with unsupervised learning for joint channel and power allocation [18] and coalition game theory combined with Dinkelbach–SCA-based power optimization for uplink NOMA systems [19]. However, mathematical optimization finds it difficult to address NP-hard problems, and convexity is hard to satisfy [20]; while reinforcement learning, despite its potential, is still limited by computing power and practical adaptability in industrial deployments [21,22,23,24].

Hybrid user is a cutting-edge concept based on CRNs; such a user has the ability to selectively access PN and SN based on the channel situation [3]. When this kind of user is in the PN, it has the same channel preference and usage rights as a normal *PU*, and its priority is higher than that of a normal *SU*; when it is in the SN, it has the same priority as an *SU* and can exist as an *SU*, and through such behavioral mode switching, the channel utilization is improved. Currently, research is underway to optimize user behavioral mode switching and linkage in CR networks. For example, in [7], the joint perceived duration and subchannel power optimization algorithm for the same *SU* in Overlay/Underlay dual-mode is investigated, but the scope of the discussion is only for single-*SU* scenarios, and there is a lack of consideration of multi-user interference and fairness. The joint optimal allocation of bandwidth + power under OM/UM switching in CR networks has been studied in the literature [8], but it lacks the consideration of IoT metrics, such as energy efficiency, and it is only for single-*PU* scenarios, with a limited degree of expansion.

In order to solve the problem of a single optimization index and low scenario expansion in the existing work in this field, this paper introduces the hybrid user concept to jointly model the system energy efficiency, *SU* average SINR, and *SU* fairness. The article adopts a heuristic optimization approach to solve the problem and proposes the Hybrid Mode-Aware Allocation with Coupled Power Control (HMAC) algorithm. The “Mode-Aware Allocation” principle refers to dynamically selecting the most suitable access mode (IM or UM) for each *SU* based on real-time channel state information, *PU* activity patterns, and interference constraints, ensuring that the allocation strategy adapts to both spectrum availability and network performance requirements. The core of HMAC is based on mode switching, an adaptive threshold, channel assignment based on the Hungarian algorithm, and a multi-stage power adjustment strategy. Among these, the Hungarian algorithm is widely used in user–subcarrier allocation and subchannel assignment. It has been proven to help reduce channel interference and improve network efficiency under different optimization conditions [25,26]. Power adjustment strategies are widely used in large-scale multiple-input multiple-output (MIMO) communication systems, and have been proven to be closely related to user fairness and network energy efficiency [27,28]. HMAC extends the concept of hybrid users by scheduling system spectrum resources, transmitter power, user space resources, channel interference, etc., in terms of channels, and focuses on the behavioral logic of *PU*s and *SU*s in the channel, which weakens the concepts of PN and SN in the CRNs, and maximizes the performance metrics adopted. HMAC, from the basis of the generalized concept of *SU*s, empowers the *SU* to choose whether to co-exist with the same channel *PU* or choose an empty channel for transmission. Interweave–Underlay Mode (I–UM) linkage is used to redefine the behavioral logic of such users. And a multi-objective optimization scheme is used to design the optimization algorithm for the system energy efficiency (EE), the Jain’s fairness index of *SU*s and the average SINR of *SU*s. In this paper, a large number of simulations are carried out to verify the advantages and robustness of the HMAC algorithm in terms of EE, Jain’s fairness index of *SU*s and average SINR for a certain *PU* fulfillment rate in terms of dimensions such as the number of *SU*, the maximum power, and the diameter of the user-generated cell.

This paper is organized as follows: Section 2 introduces the system model, including user node deployment and the channel fading model. In Section 3, the optimization problem is formulated, and a complexity analysis of the problem is performed. In Section 4, a hybrid user-based heuristic algorithm is proposed to solve the optimization problem. Section 5 presents a variety of simulations and discusses them based on experimental results. Section 6 concludes this paper and gives an outlook for future research.

## 2. System Model

**Figure 1:**
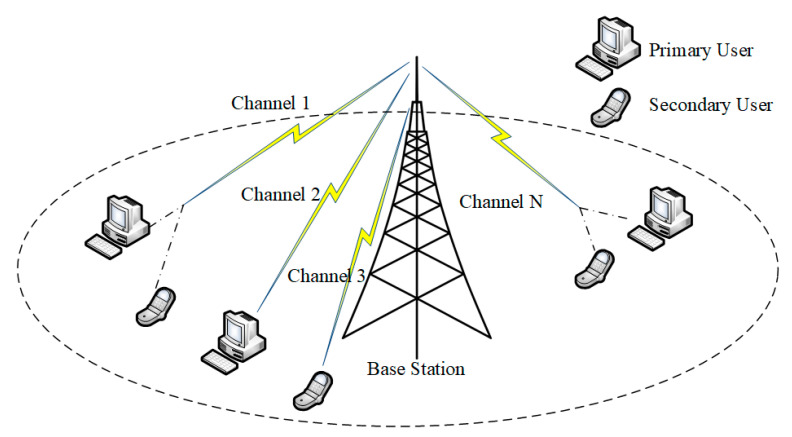
Cognitive radio network model.

As shown in Figure 1, the system model of this study focuses on the channel allocation of *PU*s and *SU*s under a CR network, in which, due to the use of the concept of mixed users, the system model does not differentiate between PNs and SNs based on the traditional CR network, but uses the channel as the basic unit, and combines the *PU*s and *SU*s among a limited number of channels. The system model is defined based on the CRN model descriptions in [1,21]. Let the total number of channels be $N_{Channel}$, the number of *PU*s be $N_{PU}$, and the number of *SU*s be $N_{SU}$. Their quantitative relationships are satisfied as

(1) $$0<N_{PU}<N_{Channel},0<N_{SU}<N_{Channel}$$

In this network, the Base Station (BS) is aware of $N_{Channel}$ channels, and at most one *PU* and one *SU* exist simultaneously in the same channel. There is no situation where two or more *PU*s or *SU*s exist in a channel at the same time.

To emulate the channel environment and spatial layout of a cognitive radio network, this paper constructs a simplified system model on a two-dimensional plane, illustrated below.

Primary Users (*PU*s): There are *N PU*s in the system, each *PU* occupies an independent channel, and the spatial locations of the *PU*s are distributed in a two-dimensional real-numbered domain as

(2) $$p_{i}^{PU}=\left[\begin{array}{l} x_{i}^{PU}\\ y_{i}^{PU} \end{array}\right]\in {\mathbb{R}}^{2},i=1,\ldots ,N$$

where $p_{i}^{PU}$ represents the coordinates of the ith *PU* in that two-dimensional space, with a horizontal coordinate of $x_{i}^{PU}$ and a vertical coordinate of $y_{i}^{PU}$.

Secondary Users (*SU*s): The system generates up to *N SU*s based on the experimental parameters. The location of the *SU*s is restricted to a smaller area, which is a concentrated cluster as

(3) $$p_{j}^{SU}=\left[\begin{array}{l} x_{j}^{SU}\\ y_{j}^{SU} \end{array}\right]\in {\mathbb{R}}^{2},j=1,\ldots ,\min(N,num_{SU})$$

where $p_{j}^{SU}$ represents the coordinates of the jth *SU* in that two-dimensional space, with horizontal coordinates $x_{j}^{SU}$ and vertical coordinates $y_{j}^{SU}$.

If $num_{SU}<N$, the corresponding *SU* is an unassigned user on the redundant channel. In addition, the system defines a center base station location $p_{BS}$ that can be used in the path loss calculation.

In this paper, it is assumed that both *PU*s and *SU*s face random small-scale fading, and their channel gains are modeled as non-negative real numbers after taking the absolute value of the Gaussian distribution, i.e., the Rayleigh fading amplitude approximation is processed as

(4) $$h_{n}^{PU},h_{n}^{SU}∼\left|N\left(0,1\right)\right|,n=1,\ldots ,N$$

In order to characterize the interference of *SU*s on *PU*s, this paper uses the free-space propagation model to construct the power gain of the interference link. According to the description of the Friis transmission equation by Rappaport, T. S. in Wireless Communications—Principles and Practice, Second Edition [29], the formula is determined as

(5) $$g_{n}^{\mathrm{int}}=\frac{G_{t}G_{r}\lambda^{2}}{{\left(4\pi d_{n}\right)}^{n_{path}}}$$

where $G_{t}$ denotes the transmitter antenna gain, $G_{r}$ denotes the receiver antenna gain, is the wavelength, $d_{n}$ the Euclidean distance between the nth *SU* and the *PU*, and $n_{path}$ is the path loss index.

The received signal-to-interference-plus-noise ratios (SINRs) of *PU*s and *SU*s on the nth channel are defined as in Equation (6) following the formulation in [1].

(6) $$\gamma_{n}^{PU}=\frac{h_{n}^{PU}p_{n}^{PU}}{g_{n}^{\mathrm{int}}p_{n}^{SU}+N_{0}},\gamma_{n}^{SU}=\frac{h_{n}^{SU}p_{n}^{SU}}{g_{n}^{\mathrm{int}}p_{n}^{PU}+N_{0}}$$

The overall system throughput is then obtained by summing the Shannon capacities of all channels allocated to the *PU*s and *SU*s, expressed in Equation (7) [30] as

(7) $$T_{sys}=\sum_{n=1}^{N}\log_{2}(1+\gamma_{n}^{PU})+\sum_{n=1}^{N}\log_{2}(1+\gamma_{n}^{SU})$$

## 3. Problem Formulation

The aim of this paper is to develop a power allocation strategy for multiple *SU*s in a cognitive network that maximizes the overall system performance without interfering with the normal communication of *PU*s. To this end, the following four categories of core performance metrics are considered in this paper, namely *PU* Satisfiability, *SU* Average SINR, Energy Efficiency (EE), and Jain’s Fairness Index. The reason for choosing these four metrics is to comprehensively weigh the system’s ability to protect the communication quality of the *PU*s while achieving the communication performance improvement of the *SU*s, as well as to take into account the optimization of the energy efficiency of the resource use and the fairness among multiple users. Among them, *PU* satisfaction is used as one of the constraints of the objective function to measure the effectiveness of the protection of the *PU*s, the *SU* average SINR reflects the service quality of the *SU*s, the energy efficiency index evaluates the benefits gained by the system under the power consumption, and Jain’s fairness index quantifies the degree of balance of the resources obtained by each *SU* to ensure that the system does not over-optimize individual users at the expense of the overall fairness.

The above four indicators are harmonized and integrated into an objective function OF of the following form:

(8) $$OF:\max\left\{\beta_{EE}\cdot \frac{EE}{EE_{\max}}+\beta_{Jain}\cdot J+\beta_{SU}\cdot \frac{{\bar{\gamma }}_{SU}}{\gamma_{\max}}\right\}$$

OF is subject to:

(9) $$s.t.\;C_{1}:\frac{N_{sat}}{N}\geq \tau$$

(10) $$C_{2}:0<p_{PU}\leq p_{\max}$$

where *C*_1_ and *C*_2_ are the constraint conditions of the optimization problem. Specifically, *C*_1_ requires that the *PU* satisfaction ratio must exceed the threshold τ and *C*_2_ ensures that the *PU* transmit power remains within the allowed range. $EE_{\max}$, $\gamma_{\max}$ are the normalization factors taken from the maximum of all the current strategy outcomes. $\beta_{EE}$, $\beta_{Jain}$, $\beta_{SU}$ are the weighting coefficients for each part of the objective function.

For EE, according to [27], it is calculated as the ratio of the system throughput to the sum of the total power of the *PU*s and *SU*s in that channel. A higher EE value indicates better system energy efficiency. The equation for calculating EE is formed by Equation (7) and total power consumption, noted as:

(11) $$EE=\frac{T_{sys}}{\sum p_{PU}+\sum p_{SU}}$$

The article uses the Jain fairness index to quantify the fairness of SINR allocation among active *SU*s. According to the study of Jain et al. [31] and Equation (6), the Jain fairness index is formed as

(12) $$J=\frac{{\left(\sum_{i\in S}\gamma_{i}^{SU}\right)}^{2}}{\left|S\right|\cdot \sum_{i\in S}{\left(\gamma_{i}^{SU}\right)}^{2}+\varepsilon }$$

where $\gamma_{i}$ denotes the SINR of the ith active *SU* and n denotes the number of sending *SU*s. Values close to 1 indicate a high degree of equity, while values close to 1/*n* indicate a high degree of inequality.

Secondly, it is observed that from Equation (8) to Equation (10), the original optimization problem OF shares the same structure as the k-Knapsack decision problem. There exists a clear correspondence between their parameters. Specifically, the k-Knapsack can be viewed as a special case of OF by treating each item as a *PU* link, mapping the item’s weight to the minimum acceptable power, the total capacity to the upper power constraint, and the target number of selected items to the number of satisfied *PU*s. Under this construction, the two problems are equivalent in the sense of determining whether a feasible solution exists. According to Kellerer et al.’s analysis of knapsack problem complexity, the k-Knapsack problem is at least NP-Complete [32]. Therefore, the original optimization problem OF is at least NP-Hard.

## 4. Optimization Algorithm Based on Hybrid User

**Figure 2:**
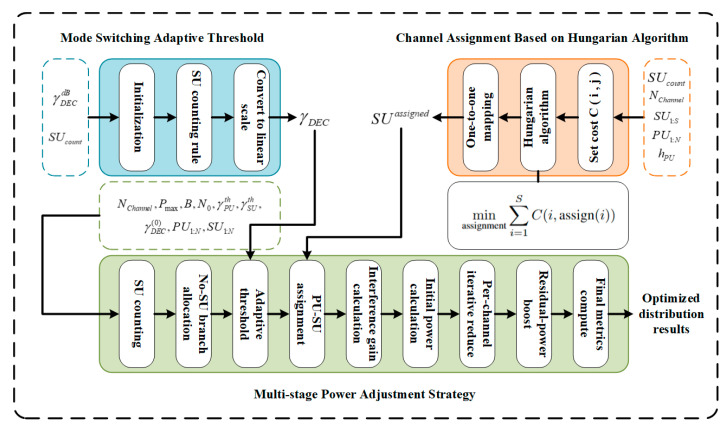
HMAC algorithm diagram.

In this section, this paper proposes a hybrid user-based heuristic algorithm, HMAC, specifically, the algorithm consists of three major parts, namely, a mode-switching adaptive threshold, channel allocation based on the Hungarian algorithm, and a multilevel power tuning strategy. Next, this paper will introduce these three parts of the algorithm in order and finally summarize the whole framework. The structure of the HMAC algorithm is illustrated in Figure 2.

A. Mode-Switching Adaptive Threshold

First, the HMAC algorithm proposed in the paper involves switching between IM and UM. These modes involve the DSA model and the SS model, which aim to make the algorithm more inclined to use the opportunistic model when the number of *SU*s is small, providing more channel resources to the *SU*s. The algorithm tends to use the *PU*–*SU* coexistence model when the number of *SU*s is large, which protects the *PU* channel resources while allocating the additional channel resources to the *SU*s for transmission as much as possible. The paper assumes that the CR network can be aware of the number of *SU*s successfully accessed in the network at this moment, which is denoted as $SU_{count}$. In addition, since this algorithm involves model switching, there exists a threshold variable $\gamma_{th}^{DEC}$ for deciding which model to use for transmission after the user assigns the channel and adjusts the power, see Algorithm 1.
**Algorithm 1** Mode-Switching Adaptive Threshold**Step/Description****Content****Input**$SU_{count}$$,\;\gamma_{DEC}^{dB}$**Output**$\gamma_{DEC}$**Initialization**$\gamma_{DEC}\leftarrow 10^{\gamma_{DEC}^{dB}/10}$**Step 1: *SU* counting rule**If
$SU_{count}\leq \frac{N_{channel}}{2}$ then
$\Delta_{dB}\leftarrow -\sigma$

Else if $SU_{count}\leq \frac{N_{channel}}{2}$
then $\Delta_{dB}\leftarrow +\sigma$

Else $\Delta_{dB}\leftarrow 0$
**Step 2: Convert to linear scale**$\gamma_{DEC}\leftarrow \gamma_{DEC}\times 10^{\Delta_{dB}/10}$**return**$\gamma_{DEC}$

Here exists a threshold adjustment value $\Delta_{DEC}$, which is represented by the congestion of *SU*s in the network when $N_{Channel}$, $SU_{count}$, and $\Delta_{DEC}$ satisfy the following relationship:

(13) $$\Delta_{DEC}=\left\{\begin{array}{l} -\sigma ,\;\mathrm{if}\;SU_{count}<\frac{N_{Channel}}{2}\\ +\sigma ,\;\mathrm{if}\;SU_{count}\geq \frac{N_{Channel}}{2} \end{array}\right\}$$

where $\Delta_{DEC}$ is used to determine the latest threshold, the old threshold is denoted by $\gamma_{th}^{DEC}$, and the new threshold is denoted by $\gamma_{th}^{DEC,new}$, which satisfies the following relation:

(14) $$\gamma_{th}^{DEC,new}=\gamma_{th}^{DEC}\times 10^{\Delta_{DEC}/10}$$

The new threshold judgment is determined by the threshold variable, which is indirectly determined by the *SU* congestion; when the number of *SU*s is very small, the threshold is lowered by σdB to encourage more *SU*s to access to the network; and when the number of *SU*s is increased, the threshold is raised by σdB to protect the channel resources of the *PU*s. With this simple channel sensing, the complexity of the model criterion can be reduced, and the computational power consumption is small.

B. Channel Assignment Based on the Hungarian Algorithm

After determining the mode-switching threshold, the algorithm has to assign channels to the *PU*s and *SU*s for which spatial relationships have been determined so far. Here, it is assumed that the *PU*s in the space have already occupied the channels separately, so for each *SU*, a cost matrix $C(i,j)\in {\mathbb{R}}^{SU_{count}\times N_{Channel}}$ is set to determine which channel the *SU* is assigned to, whether it chooses to coexist with the *PU*s in a channel or to transmit in the idle channel, see Algorithm 2.
**Algorithm 2** Channel Assignment Based on the Hungarian Algorithm**Step/Description****Content****Input**$SU_{count}$$,\;N_{channel}$$,\;SU_{1:SU_{count}}$$,\;PU_{1:N_{channel}}$$,\;h_{PU}$R**Output**$SU_{1:N_{channel}}^{assigned}$**For each **R

  **For** 
i=1
** to **
$SU_{count}$


   **For** 
j=1
** to **
$N_{channel}$

If $h_{PU}(j)=0$
then set cost C(i,j)=0

Else compute distance $d={\left\|SU_{i}-PU_{j}\right\|}_{2}$
and set $\cos t\;C(i,j)=\frac{1}{\max(d,\varepsilon )}$
**Solve linear assignment**Minimize $\sum_{i=1}^{SU_{count}}C(i,assign(i))$ using Hungarian algorithm**Obtain mapping**
One-to-one mapping: assign(i)→j
**For **j=1** to **$N_{channel}$If channel j
is matched to some $SU_{i}$
, set $SU_{j}^{assigned}=SU_{i}$

Else set $SU_{j}^{assigned}=NaN$


For determining whether a channel exists with or without a *PU*, whether it can serve an *SU* alone as an idle channel is determined by the presence of $h_{PU}$ in that channel, for C(i,j) satisfying

(15) $$C(i,j)=\left\{\begin{array}{l} 0,if\;h_{PU}(j)=0\\ \frac{1}{\max(d_{i,j,\varepsilon })} \end{array}\right\}$$

where $d_{i,j}$ denotes the Euclidean distance between the *SU* and the *PU* discussed so far. It is denoted as

(16) $$d_{i,j}={\left\|SU_{i}-PU_{j}\right\|}_{2}$$

where the distance relation between all the *PU*s and that *SU* is traversed, and when the selected *PU* has the maximum distance from the *SU*, at that point of time, the channel interference between that *SU* and the *PU* is minimum. After all the *SU*s have performed the computation of the cost matrix, the Hungarian algorithm [25,26] is performed to allocate the channel, and the Hungarian algorithm is denoted as:

(17) $$\min\sum_{i=1}^{SU_{count}}C(i,assign(i))=\min\sum_{i=1}^{SU_{count}}\sum_{j=1}^{N_{Channel}}c_{ij}x_{ij}=\min\sum_{i=1}^{SU_{count}}\sum_{j=1}^{N_{Channel}}\frac{x_{ij}}{\max({\left\|SU_{i}-PU_{j}\right\|}_{2},\varepsilon )}$$

where $x_{ij}=1$ indicates that the ith *SU* is assigned to the jth channel, and $x_{ij}=0$ indicates that it is unassigned. Where x∈χ, denotes that *x* is in the range of a two-dimensional constraint set.

With such an algorithm, after obtaining the spatial relationship between all the *SU*s and *PU*s, as well as the information about the empty channels currently in the network, the Matlab 24.2.0.2740171 (R2024b) simulation assigns a one-to-one correspondence to the *SU*s based on the cost matrix C(i,j) through the matchpairs function.

C. Multi-stage Power Adjustment Strategy

Following the mode-switching adaptive threshold and channel assignment based on the Hungarian algorithm, the proposed algorithm applies a multi-stage power adjustment strategy. This strategy is inspired by energy-efficient power control schemes in cell-free massive MIMO and heterogeneous networks, which demonstrate that adaptive power adjustment can improve energy efficiency while ensuring user fairness [27,28].

First, the model initializes the input system parameters to obtain the total number of channels, the number of *PU*s, the number of *SU*s, the maximum power, the bandwidth, the noise power, the main user SNR threshold, and the initial model-switching threshold. The maximum power is used to determine the subsequent power adjustment step, the power downward adjustment step is $\Delta_{↓}=a\%P_{\max}$ and the power upward adjustment step is $\Delta_{↑}=b\%P_{\max}$, see Algorithm 3.
**Algorithm 3** Multi-stage Power Adjustment Strategy**Step/Description****Content****Input**$N_{channel}$$,\;P_{\max}$B$,\;N_{0}$$,\;\gamma_{PU}^{th}$$,\;\gamma_{SU}^{th}$$,\;\gamma_{DEC}^{\left(0\right)}$$,\;PU_{1}^{N_{channel}}$$,\;SU_{1}^{N_{channel}}$$,\;h_{PU}$$,\;h_{SU}$**Output**$T_{sys}$$,\;\gamma_{PU}[1:N_{channel}]$$,\;\gamma_{SU}[1:N_{channel}]$$,\;p_{PU}[1:N_{channel}]$$,\;p_{SU}[1:N_{channel}]$**Constants**$\Delta_{↓}\leftarrow (a\%)\cdot P_{\max}$, $\Delta_{↑}\leftarrow (b\%)\cdot P_{\max}$**Step 1: *SU* counting**
Determine valid *SU*s S
, set $SU_{count}=\left|S\right|$
**Step 2: No–*SU* branch**
If $SU_{count}=0$: $p_{PU}=P_{\max}$ on active-*PU* channels, $p_{SU}=0$ Compute $\gamma_{PU}$ and $T_{sys}=\sum B\log_{2}(1+\gamma_{PU})$ Return**Step 3: Adaptive threshold**$\gamma_{DEC}\leftarrow \mathrm{Algorithm}\;1(SU_{count},\gamma_{DEC}^{dB})$**Step 4: *SU*–*PU* assignment**$SU^{assigned}\leftarrow \mathrm{Algorithm}\;2(PU,SU,h_{PU})$**Step 5: Interference gain**
For n=1 to $N_{channel}$: If $h_{PU}(n)=0$ or $SU_{n}^{assigned}=NaN$$,\;\mathrm{set}\;g_{\mathrm{int}}(n)=0$ Else $d={\left\|PU_{n}-SU_{n}^{assigned}\right\|}_{2}$$,\;g_{\mathrm{int}}(n)=\frac{G_{t}G_{r}\lambda^{2}}{{\left(4\pi d\right)}^{n_{path}}}$**Step 6: Initial power**Set $p_{PU}=P_{\max}$ on *PU*-present channels $p_{SU}=P_{\max}$ where *SU* assigned**Step 7: Per-channel iterative reduction**For n=1 to $N_{channel}$: If *PU* absent: $\gamma_{SU}(n)=\frac{h_{SU}(n)p_{SU}(n)}{N_{0}}$ Else if *SU* absent: $\gamma_{PU}(n)=\frac{h_{PU}(n)p_{PU}(n)}{N_{0}}$ Else: If $\frac{h_{PU}p_{PU}}{g_{\mathrm{int}}p_{SU}+N_{0}}<\gamma_{DEC}$, set $p_{SU}(n)=0$ and continue Repeat until converged: $g_{PU}=\frac{h_{PU}p_{PU}}{g_{\mathrm{int}}p_{SU}+N_{0}}$$,\;g_{SU}=\frac{h_{SU}p_{SU}}{g_{\mathrm{int}}p_{PU}+N_{0}}$ If $g_{PU}>\gamma_{PU}^{th}$: $p_{PU}-=\Delta_{↓}$ Else if $g_{PU}<\gamma_{PU}^{th}$: $p_{SU}-=\Delta_{↓}$ Else if $g_{SU}<\gamma_{SU}^{th}$ and $g_{PU}>1.2\gamma_{PU}^{th}$: $p_{PU}-=\Delta_{↓}$ Else break**Step 8: Residual-power boost**Distribute surplus power on idle-*PU* channels using classic water-filling**Step 9: Coexistence-channel enhancement**Increase $p_{SU}$ oncoexistence channels by $\Delta_{↑}$, ensuring $\gamma_{PU}\geq \gamma_{PU}^{th}$ and $p_{SU}<P_{\max}$
**Step 10: Final metrics**$\gamma_{PU}=\frac{h_{PU}p_{PU}}{g_{\mathrm{int}}p_{SU}+N_{0}}$$,\;\gamma_{SU}=\frac{h_{SU}p_{SU}}{g_{\mathrm{int}}p_{PU}+N_{0}}$ $T_{sys}=\sum B\log_{2}(1+\gamma_{PU})+\sum B\log_{2}(1+\gamma_{SU})$

The process starts by counting the number of effective *SU*s within range and storing it in $SU_{count}$. If there are no *SU*s, the system calculates the *PU* throughput and SINR directly and ends the loop. If *SU*s are present, the mode-switching adaptive threshold is executed to update itself. Next, the channel assignment based on the Hungarian algorithm allocates channels to reduce interference. For each channel, if the *PU* is idle or no *SU* exists, the interference is set to zero. Otherwise, the *SU*-to-*PU* coupling is computed based on the path loss model. After parameter setup, power initialization begins. Each *PU* and *SU* is first assigned Pmax, and iterative tuning follows based on interference. In coexistence channels, if the *PU* SINR is below the threshold, the DSA algorithm shuts down the *SU* to protect the *PU*. If the SINR is acceptable, the SS strategy adjusts power: it reduces *PU* power when the SINR is too high, reduces *SU* power when the SINR is too low, and reuses the *PU* margin to help *SU*s if the *PU* SINR is stable. Finally, idle *PU* channels are reused by *SU*s using water-filling, and *SU* power is increased by $\Delta_{↑}$ step-by-step without breaking *PU* protection. After power adjustment, SINR and throughput are recalculated.

## 5. Simulations

In this section, the paper will simulate and discuss the objective function and weights identified in Section 3, as well as the involved energy efficiency, *SU* average SINR, and Jain’s fairness index.

For user generation, the model simulates the principle of linear random distribution of *PU*s in a large area and *SU*s in a small area, by which the abstract model simulates a real scenario in a city such as a car or a bus passing by in a street or a residential area, and the whole is modeled after the rules for defining clusters of *SU*s and the two-dimensional plane of Moayedian et al. in [2]. Let the *PU*s be generated in a two-dimensional plane of length a and width b. The generation rule is linear random. The base station is located at the orthocenter of this plane and serves as the coordinate origin. The *SU* generation forms user clusters in a circular region of radius c with center coordinates (*d*,*e*), which also allows *PU*s to be generated within its boundaries, and the circular region satisfies that all the coordinates of the points within the circle are within the two-dimensional plane in which the *PU* is generated, and the center of the circle generation rule is

(18) $$c_{x}=d=R+(a-2R)\cdot u,u\sim U(0,1)$$

(19) $$c_{y}=e=R+(b-2R)\cdot v,\;v\sim U(0,1)$$

In order to reflect the advantages of this paper’s HMAC algorithm in terms of the research objectives, this paper adopts multiple baseline algorithms for simulation. The greedy model (Greedy) improves the total system throughput by randomly assigning a limited number of *SU*s to the channel and gradually decreasing the transmit power only for the *SU*s based on the initial full power configuration. In this process, the SINR thresholds for *PU*s and *SU*s are not considered, and no precise interference control is performed. The opportunistic model (Opportunistic) makes the *SU* transmit only in the channel where the *PU* is empty. The coexistence model (Coexistence) does not guarantee the minimum SINR of the *PU*, so that the *SU* and the *PU* transmit in the same channel. The Max-Power model, on the other hand, enables the *SU* and the *PU* to transmit at maximum power simultaneously, which belongs to a variant of the Coexistence model and does not consider channel interference between the two.

In terms of computational complexity, the Greedy, Opportunistic, Coexistence, and Max-Power models have relatively low complexity, typically O(M×N), or lower, where M denotes the number of available channels and N denotes the number of users. These models do not require global matching. In contrast, the proposed HMAC algorithm employs the Hungarian algorithm for channel assignment, which has a complexity of $O(N^{3})$, enabling more optimal *SU*–*PU* spatial matching at the cost of increased computation.

**Figure 3:**
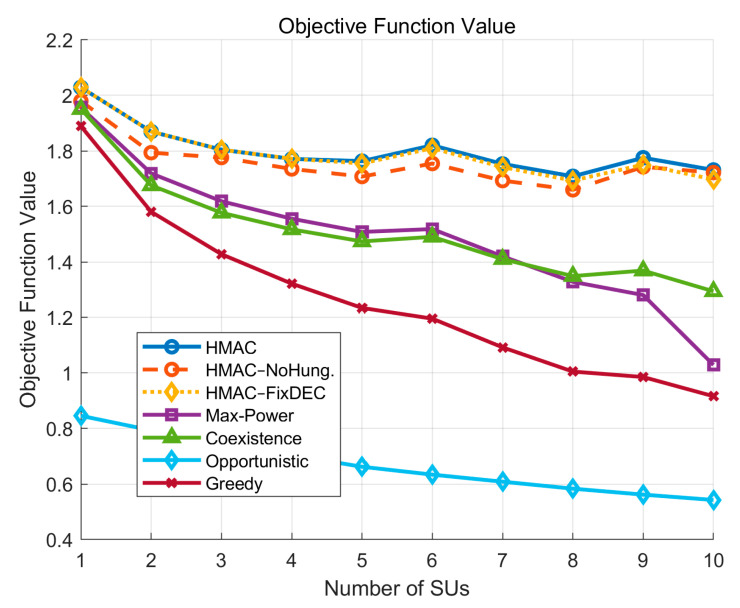
The target value varies with the number of *SU*.

The simulation parameter settings are described below, see Table 1:

First, in accordance with the node allocation rules in Section 2, the number of *SU*s varies from 1 to 10 when there are 10 *PU*s in the network. As shown in Figure 3, for the objective function, the HMAC curve is the optimization algorithm proposed in this study. Overall, the performance is relatively outstanding, with a slightly downward trend with the increase in the number of Sus, but it is not much affected by the growth of the number of *SU*s compared to other algorithms, which is due to the fact that under the framework of the HMAC algorithm, the energy efficiency of the system increases significantly with the increase in the number of *SU*s. This growth weakens the negative impact of the decrease in the average SINR, and Jain’s fairness of the *SU*s increases. Thus, the overall effect of the algorithm maintains a certain degree of robustness. For the other algorithms, compared to HMAC, they all decrease significantly with an increase in the number of *SU*s, and the Max-Power curve and the Greedy curve decrease the most, which is due to the fact that all their related performance metrics exhibit a decreasing trend.

**Figure 4:**
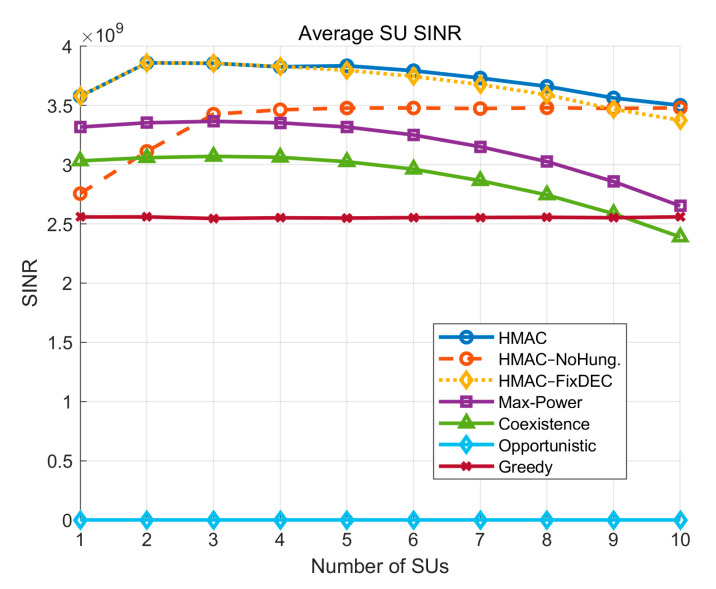
Average *SU* SINR varies with the number of *SU*s.

For the average SINR of *SU*s, according to Figure 4, the HMAC curve is at the top of the entire graph throughout the entire process. Moreover, compared with the maximum power model and the coexistence model, the robustness of the HMAC model is the highest. The difference between its maximum and minimum values is very low, and the curve changes smoothly. When the number of *SU*Ss is 5, the adaptive threshold of the HMAC mode-switching part is triggered. The weights of the coexistence strategy and the opportunistic strategy within the model change. The model is more inclined to protect the channel quality of the *PU*, thereby forcing some *SU*s to be silent within the channel. However, the *SU*s that can trigger channel coexistence with the *PU* naturally have richer channel resources. Because the *PU* can tolerate its existence in the shared channel, it indicates that these *SU*s are better in spatial position and have less interference, thereby achieving higher channel utilization efficiency. This dynamic handover mechanism, based on channel awareness and spatial topology, not only guarantees the communication quality of the *PU* but also enables the remaining active *SU*s to transmit data in a lower-interference environment, further raising the average SINR level of the *SU*s. Therefore, the HMAC model still maintains good performance stability and channel fairness in multi-user congestion scenarios, demonstrating highly robust resource scheduling capabilities. This is why HMAC outperforms HMAC–FixDEC as the number of *SU*s increases. However, the other curves all show a significant downward trend as a whole. Among them, the value of the Opportunisitic curve remained at 0 throughout because the channel was fully occupied by *PU*s at this time and *SU*s did not have the channel conditions for normal transmission.

**Figure 5:**
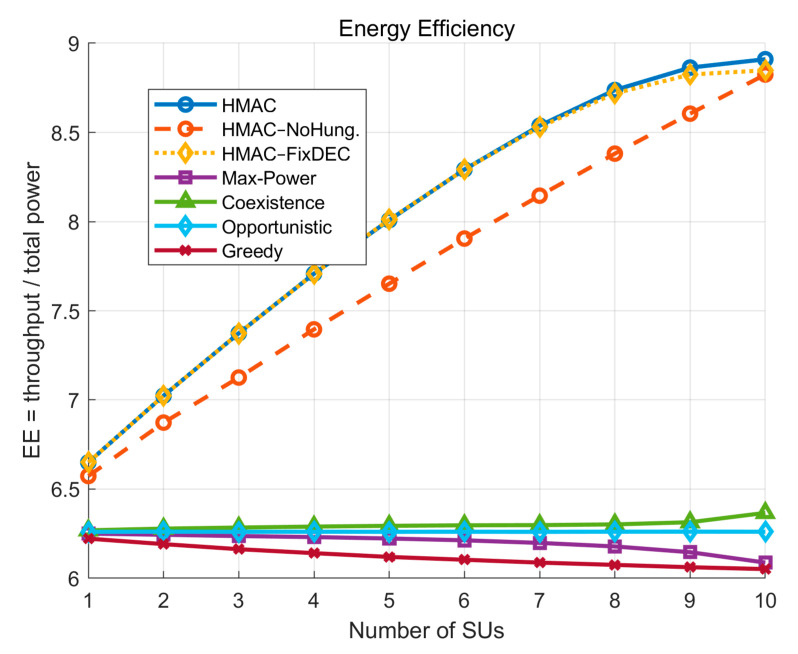
System energy efficiency varies with the number of *SU*s.

In terms of EE comparison, according to Figure 5, the HMAC model consistently maintains the highest energy efficiency value over the entire range of the number of *SU*s. The energy efficiency increases steadily with the increase in the number of *SU*s, showing good scalability. This shows that HMAC is able to effectively improve the overall EE of the system while guaranteeing the communication quality of the primary users (*PU*s). Compared with the same series of HMAC–NoHung, HMAC further benefits from the Hungarian algorithm’s optimization of *SU*–*PU* spatial matching to achieve the optimal resource allocation in multiuser scenarios. The HMAC and HMAC–FixDEC curves almost coincide when the number of subusers is small, which is due to the fact that all *SU*s preferentially take up the opportunistic channel in low-density scenarios with plenty of free *PU* channels. Both algorithms are able to achieve optimal power utilization efficiency. This is because in low-density scenarios where there are plenty of free *PU* channels, all *SU*s preferentially occupy opportunistic channels, and the results of the two algorithms are basically the same in terms of power allocation and threshold setting. HMAC further benefits from the Hungarian algorithm’s optimization of the *SU*–*PU* spatial matching and the adaptive regulation mechanism of the dynamic judgment threshold, so that it still achieves the optimal resource allocation in multiuser scenarios. In contrast, the energy efficiency of the traditional models, such as Max-Power, Opportunistic, and Greedy, basically remains unchanged or slightly decreases, reflecting the lack of effective interference control and resource coordination mechanisms when the user density increases. Taken together, the HMAC model has a significant advantage in energy efficiency, reflecting its high resource sensitivity and scheduling robustness.

**Figure 6:**
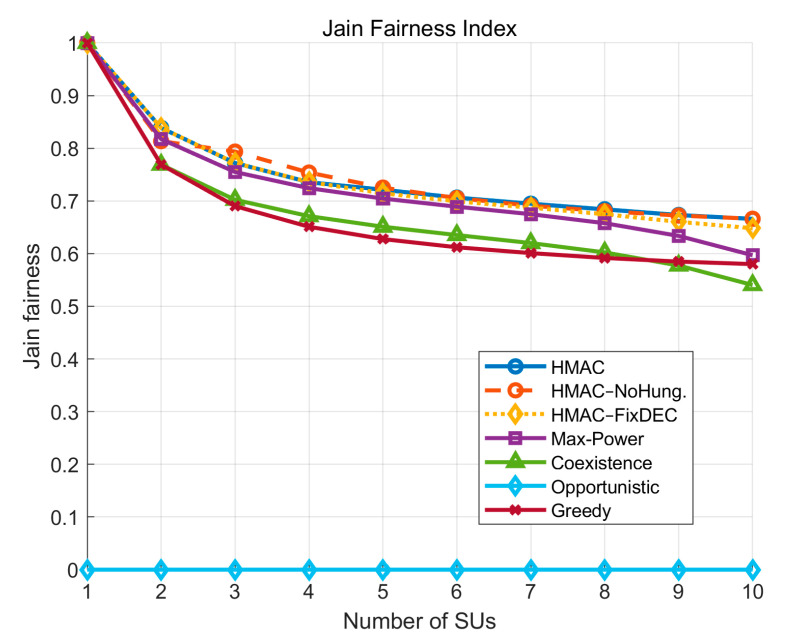
Jain’s fairness index varies with the number of *SU*s.

In the evaluation of Jain’s Fairness Index, according to Figure 6, the performance of all the curves decreases gradually with the increase in the number of *SU*s. Still, the three models of the HMAC series (HMAC, HMAC–NoHung, HMAC–FixDEC) have the highest values throughout the process and show higher fairness. Among them, HMAC–NoHung has even slightly higher fairness than the original HMAC model with Hungarian assignment when the number of *SU*s is small (2 to 4). This is because, in low-density scenarios, the random channel assignment may, by chance, circumvent the strong interference paths between *SU*s and *PU*s, allowing some *SU*s to access the channel at a lower cost and thus obtain a higher transmission efficiency. Since the Jain’s fairness index is calculated based on active *SU*s only, and those *SU*s that are forced to be silent are not counted in the fairness statistics, those *SU*s that are not silenced and are randomly assigned better channels increase the overall fairness index. This localized advantage due to randomness is amplified in small-scale networks, which is reflected in the slightly better curve in the absence of the Hungarian mechanism. However, as the number of *SU*s increases, HMAC gradually returns to its advantage in fairness performance by virtue of its interference-sensing and global matching capabilities.

**Figure 7:**
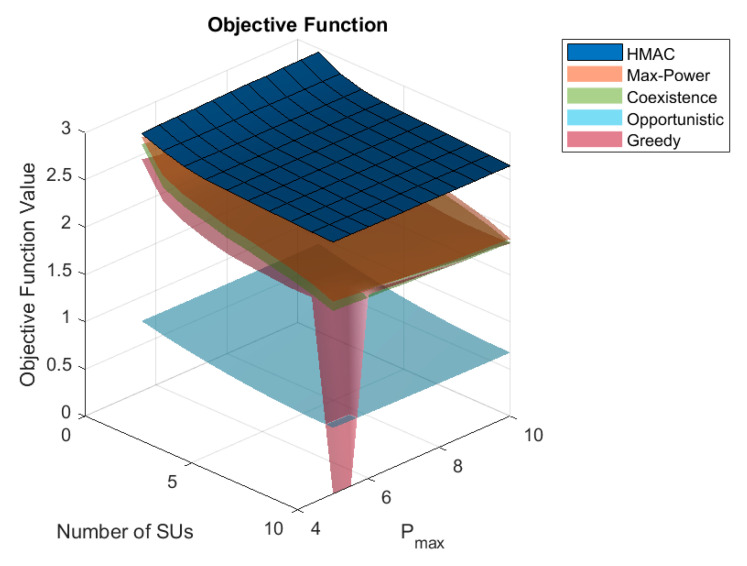
Three-dimensional relationship between target value, maximum power, and number of *SU*s.

In order to observe the relationship between the user’s maximum power, according to Figure 7, the number of *SU*s, and the optimization target, this paper presents a joint plot of these parameters. In this 3D plot, the *x*-axis represents the maximum power value, the *y*-axis represents the number of *SU*s, and the *z*-axis represents the objective function. The higher value of the target indicates better performance of the algorithm. From this figure, it can be seen that the HMAC scheme has the best performance across the range of variation of the Pmax value and the number of *SU*s. The plane formed by HMAC is more horizontal, which indicates the robustness of the scheme compared to other schemes.

**Figure 8:**
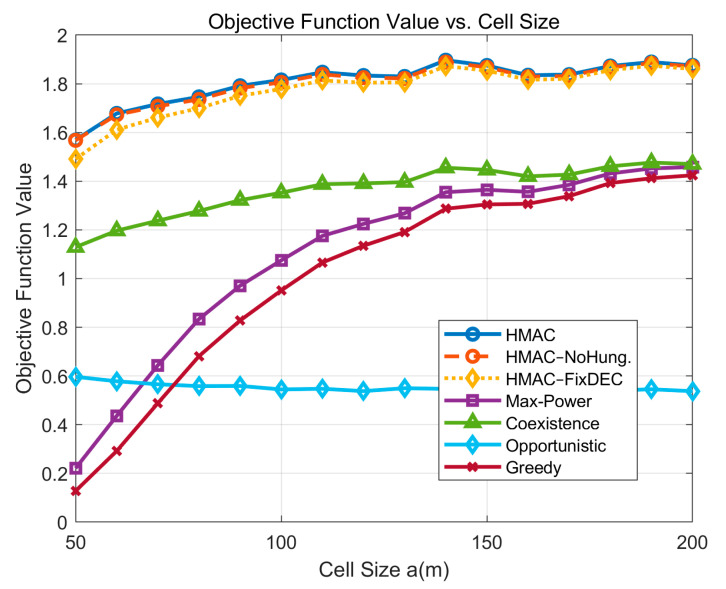
Cell size varies with the number of *SU*s.

For the cell size, this experiment simplifies the 2D plane generation rule by making L = W = a = b so that the surfacegenerated by the *PU* is a square plane. From the simulation results, according to Figure 8, it can be seen that the HMAC model still resides in the uppermost part of the map, and the performance advantage is obvious. In the HMAC series of curves, the smaller the cell diameter, the more obvious the advantage of the algorithm proposed in this paper. When the cell diameter becomes larger, the performance of the three curves tends to be consistent. This is because the cell range becomes larger, the channel interference between *SU*s and *PU*s is reduced, and the advantage of the channel allocation algorithm and adaptive threshold is no longer obvious. However, the HMAC series algorithms have an overall trend of better performance with a larger cell range.

**Figure 9:**
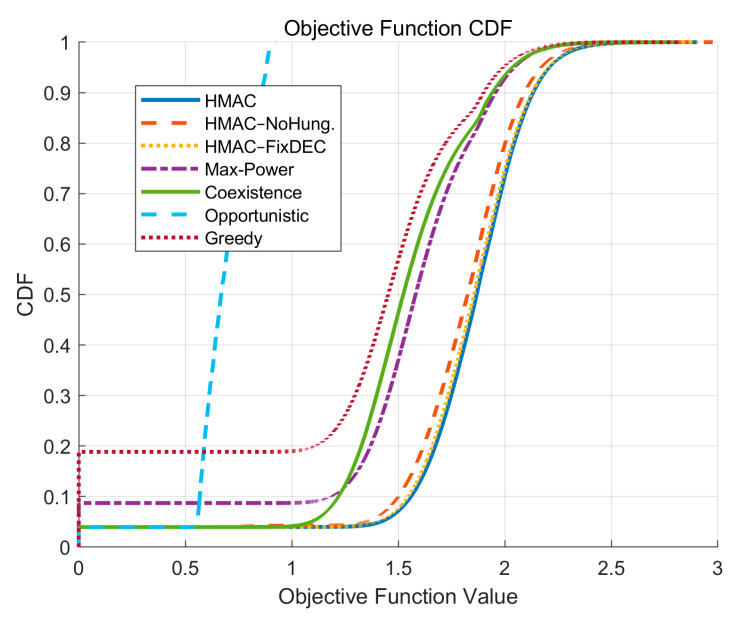
CDF curves for target values.

In the Cumulative Distribution Function (CDF) curve of the objective function, according to Figure 9, the HMAC curve is always on the rightmost side of all the compared algorithms, which indicates that, under the same level of probability accumulation, the Target Value achieved by the HMAC model is significantly higher than that of other algorithms. This not only verifies that the HMAC model has stronger convergence stability in the optimal solution space, but also reflects its superior global performance distribution in large-scale simulations.

In addition, the two variants of HMAC (HMAC–NoHung and HMAC–FixDEC) also follow and significantly outperform the traditional strategies (e.g., Greedy and Opportunistic), with the distribution of the Greedy strategy shifted to the left, which indicates that its probability of generating high-quality objective values is much lower than that of HMAC. This right-skewed distribution indicates that the HMAC model is not only more stable on average but also has a better global performance distribution in large-scale simulations. This right-skewed distribution property suggests that the HMAC model is not only superior on average but also more robust and guarantees upper-bound performance, which maintains high objective function values even under unfavorable network states.

Table 2 and Figure 3, Figure 4, Figure 5, Figure 6, Figure 7, Figure 8 and Figure 9 show that the proposed HMAC algorithm achieves the best overall performance, with the highest average objective function value (1.81), *SU* SINR (3.72), energy efficiency (8.01 bit/Joule), and fairness (0.75). These results demonstrate that HMAC can effectively balance throughput, interference control, and resource allocation, ensuring both *PU* protection and *SU* performance. The HMAC–NoHung and HMAC–FixDEC variants perform slightly worse, confirming the benefit of Hungarian-based allocation and adaptive DEC threshold switching. In contrast, Max-Power and Coexistence suffer from lower SINR and objective values due to either excessive interference or inefficient spectrum utilization, while Opportunistic yields the lowest overall performance because of its overly conservative access strategy. Across varying *SU* numbers and cell sizes, HMAC consistently maintains a clear advantage, and its CDF curve indicates greater stability, reliability, and robustness than all baselines.

## 6. Conclusions

In this study, we discuss the complexity of the joint optimization problem of Energy Efficiency (EE), Jain’s fairness index, and *SU* average SINR. After proving it to be an NP-hard problem, an HMAC algorithm is proposed on the theoretical basis of CR networks based on the concept of hybrid users, linking the Interweave and Underlay modes of CR networks. The model proposed in the article has major innovations, such as a switching adaptive threshold, channel allocation based on the Hungarian algorithm, and a multilevel power adjustment strategy, and the model excels in the performance comparison of many baselines by virtue of the above innovations. Based on the coexistence relationship between users in the same channel and the multi-channel structure of the network described in Section 2, the HMAC algorithm performs exceptionally well in CRNs with more users and larger cells. It is of great significance for future research on basic mode switching and linkage optimization with the help of CR networks. For the optimization problem established in this paper, the performance of the HMAC model is maintained at a high and smooth level, which is significantly better than the rest of the strategies. In contrast, the traditional models, such as Greedy, Max-Power, and Coexistence, show an obvious decreasing trend, and the final target performance index is lower than that of HMAC by about 25–45%. Meanwhile, the weaker versions of HMAC (e.g., HMAC–NoHung and HMAC–FixDEC) maintain overall superior performance despite slight fluctuations in some scenarios, further verifying the effectiveness of the Hungarian allocation and adaptive thresholding mechanisms in enhancing the comprehensive system performance. This indicates that the proposed HMAC model has a robust and outstanding performance in the co-optimization of multiple performance metrics.

This research is closely correlated with the development of next-generation base stations for CRNs, as the proposed HMAC algorithm depends on timely and accurate spectrum state updates, rapid and precise threshold adaptation, and frequent channel reconfiguration to achieve the joint optimization of multiple performance metrics. The envisioned advanced base stations, with enhanced sensing accuracy, reduced latency, and improved computational and control capabilities, would provide the necessary hardware support to execute HMAC’s high-complexity tasks in real time. By enabling faster spectrum sensing, dynamic decision-making, and efficient resource reallocation, such base stations would create the optimal operating environment for HMAC-like cooperative optimization algorithms, ensuring their robust and scalable performance in large-scale CRN deployments.

## Figures and Tables

**Table 1:** Parameters and settings.

Parameter Type	Parameter Setting
Number of channels ($N_{Channel}$)	10
Number of Users ($N_{PU}=N_{SU}$)	10
Maximum power ($p_{\max}$)	5 W
*PU* SINR threshold ($\gamma_{PU}^{th}$)	87 dB
Two-dimensional plane length (a)	100 m
Two-dimensional plane width (b)	100 m
Secondary user cluster radius (c)	5 m
Antenna gain ($G_{t}=G_{r}$)	1
Wavelength (λ)	$\lambda =\frac{c}{f}=\frac{3\times 10^{8}}{2.4\times 10^{9}}\approx 0.125m$
Path loss index ($n_{path}$)	3

**Table 2:** Comparison of average performance indicators for each algorithm.

	Average Target Value	Average *SU* SINR $\left(1\times 10^{9}\right)$	Average Energy Efficiency (bit/Joule)	Average Jain Fairness Index
HMAC	1.81	3.72	8.01	0.75
HMAC–NoHung	1.76	3.37	7.75	0.74
HMAC–FixDEC	1.78	3.65	7.98	0.74
Max-Power	1.50	3.16	6.20	0.73
Coexistence	1.51	2.88	6.30	0.68
Opportunistic	0.67	0	6.26	0
Greedy	1.27	2.55	6.12	0.67

## Data Availability

The original contributions presented in this study are included in the article. Further inquiries can be directed to the corresponding author.
